# The relationship between nighttime exercise and problematic smartphone use before sleep and associated health issues: a cross-sectional study

**DOI:** 10.1186/s12889-024-18100-0

**Published:** 2024-02-23

**Authors:** Yuqin Su, Hansen Li, Sijia Jiang, Yaqi Li, Yun Li, Guodong Zhang

**Affiliations:** 1 Institute of Sport Science, College of Physical Education, Southwest University, Chongqing, China; 2 College of Physical Education, Chongqing University of Posts and Telecommunications, Chongqing, China; 3 International College, Krirk University, Bangkok, Thailand

**Keywords:** Nighttime Exercise, Sleep quality, Smartphone addiction, Mental Health

## Abstract

**Objective:**

Physical exercise has the potential to mitigate addictive behaviors and relevant health issues. However, the nighttime exercise has not been studied regarding this research topic. This study aims to explore the association between nocturnal physical exercise and problematic smartphone use before sleep, as well as related health issues.

**Methods:**

To explore the association between nighttime physical exercise and problematic smartphone use before sleep as well as related health issues, we conducted a cross-sectional survey among 1,334 college students. Their daily exercise behaviors (including timeframe, rationale, frequency, and duration), smartphone use before sleep, sleep quality, smartphone addiction, anxiety, and depression were measured by questionnaires. The associations were assessed using generalized linear models.

**Results:**

Our findings indicate that nearly 70% of participants chose to perform exercise at nighttime. Among these individuals who exercised at nighttime, the frequency and duration of nighttime exercise were significantly associated with decreased probabilities of smartphone use before sleep. Additionally, the frequency and duration of nighttime exercise were associated with lower levels of smartphone addiction and anxiety disorders.

**Conclusion:**

Nighttime Exercise behaviors can effectively reduce sleep delays caused by problematic smartphone use before bedtime. These findings contribute to understanding the potential effects of nighttime exercise on problematic smartphone use and relevant health issues. Future research should employ more precise methodologies to examine these associations.

## Introduction

In the era of Internet, getting online has become an indispensable part of modern life. Smartphones, serving as conduits for internet access, have brought many conveniences for both work and leisure [1]. However, Excessive or unregulated use of smartphones may precipitate Internet addiction, also termed as “pathological Internet use.” In some early academic publications, Internet addiction was conceptualized as a form of impulse control disorder [2], whereas subsequent discourse has defined it as a persistent overuse of the Internet by individuals who have lost the capacity for self-regulation of their Internet use, culminating in compulsive Internet usage behaviors [3, 4]. This definition underscores a gradual loss of control, leading to a pervasive pattern of Internet engagement that persists despite awareness of the negative consequences and disruptions to daily life. The excessive and unhealthy use of smartphones has been underlined by the World Health Organization as a growing public health issue [5]. Numerous studies indicate a prevalent rate of smartphone addiction among the student population in higher education institutions [6–9]. As of 2017, the incidence of internet addiction among university students has escalated to an alarming 30% [10].

Problematic smartphone use often co-occurs with a range of mental health disorders, including but not limited to depression, anxiety, and stress [11, 12]. Among the multitude of concerns related to smartphone use, the use of these devices in bed before sleep has recently garnered scholarly attention. This behavior is a pivotal factor in the onset of sleep delays and disturbances among adolescents [13, 14]. Particularly, the use of smartphones while lying in bed in a dark environment poses substantial risks to visual health and sleep quality, and can lead to complications such as cervical pain, poor sleep quality, and subsequent daytime fatigue [15, 16]. These adverse impacts may further exacerbate negative emotional issues, such as depression [17, 18].

The role of physical exercise in altering behavioral outcomes has gained more attention. Known benefits of physical exercise include mitigating addictive behaviors, such as alcohol abuse, smoking, and food addiction [19–21]. Some recent studies indicate that individuals engaged in higher levels of physical activity tend to exhibit lower levels of smartphone usage [22–25], which brings the idea of using physical activity or exercise to treat smartphone addiction [26, 27]. The negative association between physical activity and smartphone use can be explained by the following aspects. According to the Mood Enhancement Theory, physical activity ostensibly reduces reliance on smartphones by enhancing subjective well-being and self-efficacy [22, 28–30]. Furthermore, although some smartphone use can occur while performing physical activity (e.g., watching short videos on a stationary bike), smartphone use is generally associated with sedentary [31, 32]. Therefore, according to the isotemporal substitution model (ISM), physical activity, as the opposite of sedentary, may occupy the time that can be otherwise spent on smartphone usage [33, 34]. Additional investigations have also demonstrated that physical activity, as an intervention strategy, can effectively change adverse psychological health outcomes and ameliorate social dysfunction [20, 35, 36]. Moreover, it is commonly accepted that physical activity contributes to improved sleep quality and other health indices [37, 38].

Although the health benefits of physical exercise have garnered considerable attention, the effect of nighttime exercise is rarely studied. In contrast to the more standardized work time regimes in Western developed countries, Asian nations, including China, often exhibit extended working hours and a prevalent culture of overtime [39, 40]. The cultures, values, societal structures, and Human Resource Management (HRM) practices of Asian countries may uniquely influence individuals’ work and life experiences [39, 41, 42]. For example, the work schedule named “996” (defined as six days of work per week, with daily hours from 9 a.m. to 9 p.m.) has become very common in China [43, 44]. In the realm of education, Asian students face a more substantial academic burden and a higher frequency of extracurricular tutoring compared to their Western counterparts [45]. This situation leads to a scenario where the majority of the population in many Asian countries, including China, tend to engage in leisure and physical activities primarily during the nighttime. This situation underscores the value of studies on nighttime activities, such as nighttime physical activity.

Previous research has indicated disparities between daytime and nighttime exercise in terms of health benefits [46]. Certain scholars have posited that nighttime physical activities might inhibit the secretion of melatonin and cause an elevation in body temperature, both of which can interfere with the sleep mechanism and consequently lead to sleep onset delay [47–49]. Specifically, as a critical hormone regulating the sleep-wake cycle, melatonin may be suppressed during intense nocturnal exercise, disrupting its normal rhythm [50, 51]. Concurrently, engaging in strenuous activities can result in sustained higher body temperatures, while the natural initiation of sleep is usually accompanied by a drop in core temperature [52]. The American Sleep Guidelines also caution against excessive physical activities at night [53]. Despite these concerns, there are also studies indicating that nighttime physical activity or exercise may not affect sleep [54–56] and might even improve sleep quality [57]. It’s worth noting that, like many developed countries, the Chinese government considers insufficient physical activity as a significant threat to public health. The potential health benefits of nighttime physical activity may vary from its daytime counterpart, but its role in weight control [58] and emotional regulation [59] can be possibly retained. Therefore, this necessary nighttime physical activity may have more bright sides than dark sides.

In addition, nighttime lighting may contribute to an extra positive experience during exercise. Concerns about the harm of artificial light at night, such as that from lighting and electronic devices like smartphones, have garnered significant attention—LED screens on phones, for example, may affect melatonin [60] secretion, disrupt circadian [61] rhythms, and lead to vision [62] impairment. Nevertheless, nighttime artificial light also holds considerable benefits, even more than its [63] harms. Firstly, it provides people with the opportunity for nighttime exercise, and outdoor street lighting may offer a comfortable light environment, allowing participants to enjoy a restorative experience during physical [64] activity. Furthermore, considering the well-documented stress-reduction capabilities of exercise [65], we hypothesize that nighttime physical exercise may mitigate reliance on other stress-reduction measures like smartphone use before sleep, thus reducing other subsequent health issues.

Despite these assumptions, empirical evidence remains relatively absent. Hence, our study aims to explore the associations between nighttime exercise and a range of health outcomes, including smartphone use before sleep, smartphone addiction, sleep quality, and related mental health issues. Specifically, we hypothesize that:

H1: Nighttime exercise behaviors are negatively associated with smartphone use before sleep (while in bed) and its duration.

H2: Nighttime exercise behaviors are negatively associated with sleep delay due to smartphone use.

H3: Nighttime exercise behaviors are positively associated with sleep quality.

H4: Nighttime exercise behaviors are negatively associated with smartphone addiction.

H5: Nighttime exercise behaviors are negatively associated with mental health issues.

## Materials and methods

### Study design and participants

A cross-sectional survey was conducted in March 2023 in the urban area of Chongqing, targeting university students enrolled in institutions within the city. This study was approved and supervised by the Ethics Review Committee of the College of Physical Education at Southwest University (code: SWU-PE-20230310). Participants were recruited from six universities in Chongqing through instructors within their respective Departments of Physical Education. Data collection was facilitated either via digital platforms or paper-based questionnaires, depending on the context and preferences of the participants. Prior to the commencement of the survey, subjects were briefed on the overarching research theme, although specific research questions were not disclosed. Informed consent forms were signed by participants before initiating the survey.

A total of 1,334 responses were received, consisting of 985 paper-based and 349 digital questionnaires. Following the exclusion of incomplete submissions (*n* = 13) and those failing manual validation tests to ensure diligent completion (*n* = 6), 1,315 qualified questionnaires were retained for subsequent analysis.

### Instruments and measurements

#### Time and rationale for engaging in physical exercise

To investigate the timeframe of physical exercise, respondents were queried with the following question: “When do you usually engage in physical exercise?” Response categories included: “I usually exercise during daytime hours,” “I usually exercise after dark,” or “I do not engage in physical exercise.” According to data from “Timeanddate,” a global resource for solar times (https://www.timeanddate.com/), the average sunrise time in Chongqing’s main urban area for March is 7:19 am, and sunset occurs at 6:53 pm. Therefore, we defined the daytime exercise as 7:20 am to 6:50 pm, and the period for nighttime exercise should be after 7:00 pm. Additionally, respondents were required to briefly elucidate their primary rationale for exercising either during the daytime or nighttime, with answers collected via open-ended boxes.

#### Frequency and duration of physical exercise

Participants were asked, " In the recent month, how many times per week on average do you engage in physical exercise during your preferred time (daytime or nighttime)?” Answers were documented using a 7-point scale (1 = once a week or less; 2 = twice a week; 3 = thrice a week; 4 = four times a week; 5 = five times a week; 6 = six times a week; 7 = almost daily). Questions pertaining to the duration of each exercise session were posed as: “Approximately how long does each exercise session last during your preferred time?” Responses were captured on a 5-point Likert scale ranging from 0 to over 120 min, with 30-minute intervals. Scores for frequency and duration were multiplied to yield the total weekly exercise time.

#### Smartphone use in bed before sleep

Two questions were designed to probe the prevalence of smartphone use in bed before sleep among college students. The first query was, “Do you usually use a smartphone while lying in bed before sleep?” Responses were coded as 0 = no and 1 = yes. The second question examined the duration of this activity, asking, “How much time do you typically spend using a smartphone while lying in bed before sleep?” Answers were captured via an 11-point Likert scale ranging from 0 to over 90 min, with 10-minute intervals.

#### Sleep delay due to smartphone use

To investigate the issue of sleep delay due to smartphone use, the question posed was: “Did using a smartphone last night cause you to sleep later than you intended, planned, or desired?” Responses were coded as 0 = no and 1 = yes.

#### Sleep quality

Drawing upon existing single-item sleep scales, an 11-point numeric rating scale was used to measure overall sleep quality, ranging from 0 (best sleep) to 10 (worst sleep) [66].

#### Smartphone addiction

The Smartphone Addiction Scale Brief Version in Chinese (SABAS) was employed to assess the risk of smartphone addiction among respondents [67]. Each question was rated using a 6-point Likert scale (1 = strongly disagree; 6 = strongly agree). The total score was computed by summing the individual item scores (ranging from 1 to 6 points), with higher scores indicating greater degrees of addiction. The scale demonstrated high reliability in our sample (Cronbach’s α = 0.80).

#### Anxiety symptoms

Anxiety levels over the past month were measured using the validated Chinese version of the General Anxiety Disorder 7-item (GAD-7) scale [68]. Responses were captured via a 4-point Likert scale for each question (0 = not at all; 3 = nearly every day). Total scores could be interpreted to diagnose anxiety symptoms. The scale showed high internal consistency in our sample (Cronbach’s α = 0.92).

#### Depressive symptoms

Depressive status over the last month was assessed using the Chinese version of the Patient Health Questionnaire-9 (PHQ-9) [69]. Responses were captured on a 4-point Likert scale (0 = not at all; 3 = nearly every day). Again, high internal reliability was observed in our sample (α = 0.90).

#### Sociodemographic characteristics

The gender of the participants was dichotomized into two groups (1 = male; 2 = female). Age categories were divided into six brackets (16–18, 19–21, 22–24, 25–27, 28–30, 31–33). Educational levels were classified into four groups (1 = Associate Degree; 2 = Bachelor’s Degree; 3 = Master’s Degree; 4 = Doctoral Degree). Monthly family income was categorized into three levels (< 5000 RMB, 5000–10,000 RMB, > 10,000 RMB).

### Statistical analysis

#### Internal reliability of the instrument

The internal reliability of the questionnaire was assessed using Cronbach’s alpha coefficient. A Cronbach’s alpha value greater than 0.70 is deemed acceptable for research purposes.

#### Jieba segmentation

In this study, the Chinese word segmentation is implemented using a commonly adopted segmentation technique - the Jieba segmentation method [70]. The fundamental principle involves constructing a prefix dictionary to segment the input sentences, subsequently generating all potential segmentation possibilities. By establishing a directed acyclic graph and employing dynamic programming algorithms, the method calculates the most probable path, thereby facilitating the segmentation function as a statistical-based approach. This segmentation technique not only enables word segmentation through its inherent algorithm but also allows for the integration of an external dictionary to enhance segmentation accuracy. In addition to segmentation, the jieba method also provides functionalities for keyword extraction and part-of-speech tagging in documents.

#### Correlation analysis

Various measures, including Spearman’s rank-order correlation for continuous variables, Phi correlation for binary variables, and point-biserial correlation for binary and continuous variables, were employed to discern the general patterns of association among the core variables.

#### Generalized linear models

Generalized Linear Models (GLMs) were utilized for assessing associations between nighttime exercise and health outcomes. Prior to analysis, the sample was segmented to focus on individuals who exercise during nighttime. The nighttime exercise items (e.g., frequency and duration) were treated as predictors. The sociodemographic characteristics, including gender, age, educational attainment, and family income, were incorporated as covariates to adjust the models. Linear and binary logistic functions were selected for continuous and binary outcomes, respectively. A p-value of < 0.05 was regarded as statistical significance.

## Results

### Participant characteristics

The characteristics of the participants are summarized in Table 1. The sample included 623 males and 692 females, with individuals aged between 19 and 21 years comprising 49.4% of the total sample. All participants were college students, with undergraduate students making up 76.8% of the sample. Nearly half (49.1%) of the participants reported a monthly family income ranging from ¥5,000 to ¥10,000. Notably, over 96% of the surveyed individuals reported to usually use smartphone before sleep, and more than half reported to have experienced sleep delay due to smartphone use.

**Table 1:** Participants’ characteristics

Variable	Category	Mean (standard deviation)	Percentage
Gender	male	-	47.4%
	female	-	52.6%
Age	16–18	-	12.2%
	19–21	-	49.4%
	22–24	-	28.8%
	25–27	-	8.4%
	28–30	-	0.7%
	31–33	-	0.4%
Education	junior college education	-	0.7%
	undergraduate course	-	76.8%
	master	-	20.8%
	doctor	-	1.7%
Monthly family income	Below 5000	-	21.8%
	5000–10,000	-	49.1%
	Above 10,000	-	29%
Smartphone Use in Bed before sleep	-	-	96.1%
Sleep Delay Due to Smartphone Use	-	-	62.4%
Physical Exercise	Exercise frequency (times)	2.77(1.60)	-
	Single duration (hours)	1.87(0.99)	-
	Weekly duration (hours)	5.65(5.46)	-
Duration of smartphone use before sleep	-	5.31(2.69)	-
Sleep quality score	-	3.95(2.28)	-
Smartphone Addiction score	-	17.88(5.26)	-
Anxiety	-	4.69(4.43)	-
Depression score	-	5.07(4.54)	-

### Proportion of individuals engaging in evening exercise

As indicated in Table 2, among the surveyed population, 22% of Chinese students exercise during the day, and 69.4% reported to conduct physical exercise during nighttime.

**Table 2:** Proportion of individuals engaging in evening exercise

Category	Number	Total	Percentage
Exercise during the daytime	289	1315	22%
Nighttime exercise	913	1315	69.4%
Never engage in physical exercise	113	1315	8.6%

### Primary reasons for opting for nighttime exercise

**Fig. 1:**
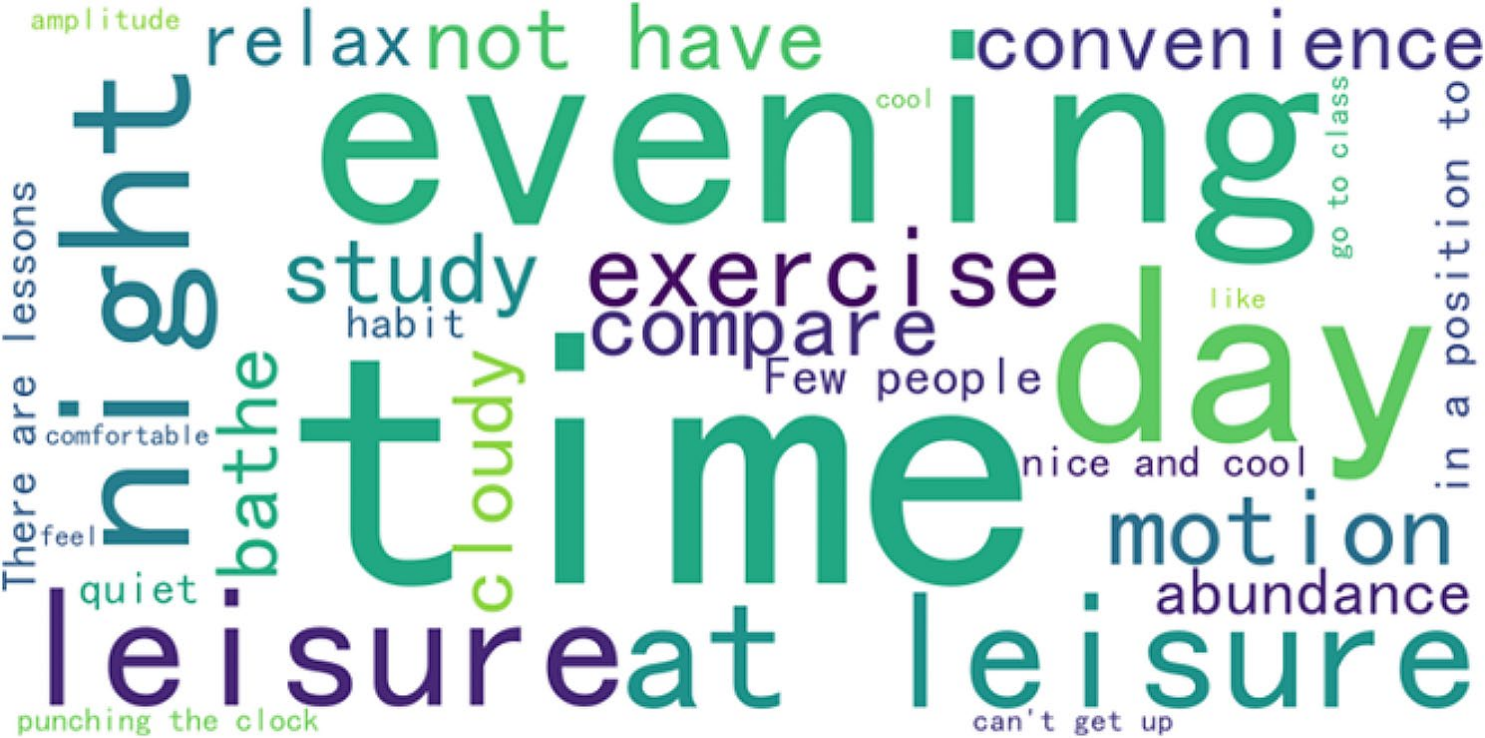
The main reasons for choosing night exercise

**Fig. 2:**
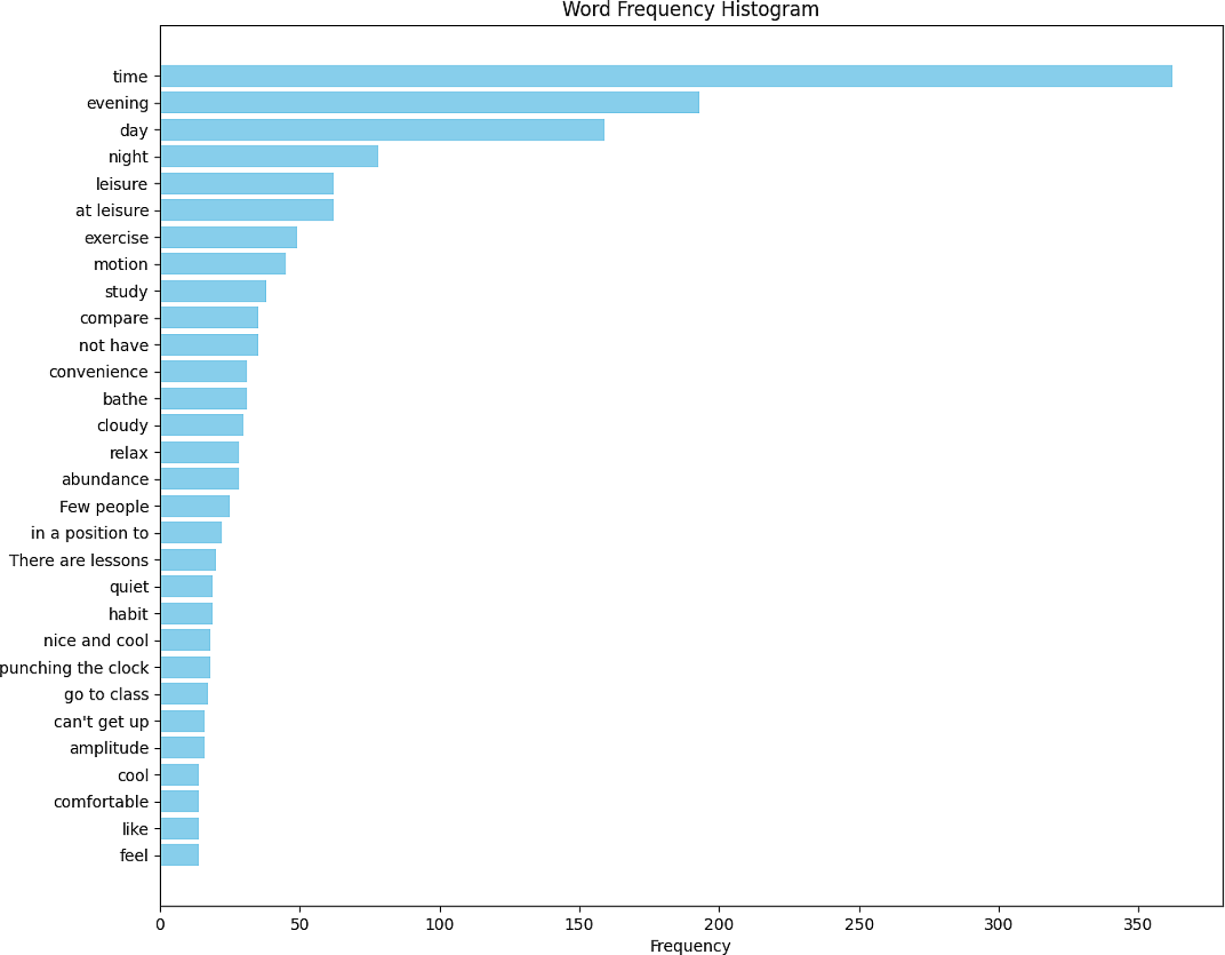
Night exercise primary reasons frequency histogram

Word clouds rendered by Python provide a visual representation of the focus and themes of interest, with the size of the words within the cloud denoting the frequency of their occurrence in the text. Following segmentation using the jieba method, and the integration of a stop-word dictionary alongside custom stop-words to eliminate meaningless words, visualization of the vocabulary is achieved. This process enhances the interpretive clarity and thematic emphasis of the textual data. Figure 1 demonstrates the top 30 keywords regarding the rationale for exercise during nighttime. Figure 2 displays a histogram of the top thirty keywords. The predominant reason for choosing to exercise at night was the availability of free time exclusively in the evening hours. This was followed by habit and the perception that nighttime exercise offered a more relaxed setting. The last mentioned reasons included the belief that exercising during nighttime aids in sleep quality and serves as a countermeasure to daytime sun exposure.

### Correlations among core variables in the nighttime exercise population

As indicated in Table 3, within the population engaged in nighttime exercise, there is a significant negative correlation between the frequency of nighttime exercise and several parameters related to smartphone use and mental health. Specifically, a higher frequency of nighttime exercise was associated with lower likelihood of smartphone use before sleep (rho = -0.088**), shorter duration of smartphone use before sleep (rho = -0.166**), and decreased likelihood of sleep delay due to smartphone use (rho = -0.117**). Additionally, a higher frequency of nighttime exercise was negatively correlated with poorer sleep quality scores (rho = -0.068*), smartphone addiction (rho = -0.165**), anxiety (rho = -0.142**), and depression (rho = -0.074*).

Furthermore, participants with a longer overall duration of nighttime exercise generally demonstrated lower probabilities of smartphone use before sleep (rho = -0.099**) and shorter duration of smartphone use before sleep (rho = -0.093**), and decreased likelihood of sleep delay due to smartphone use (rho = -0.081*). Participants with a longer overall duration of nighttime exercise also exhibit a negative correlation with smartphone addiction (rho = -0.160**) and anxiety (rho = -0.105**).

**Table 3:** Correlations among core variables

Variables		1	2	3	4	5	6	7	8	9	10	11	12	13	14
	1.Gender	1													
	2.Age	0.190**	1												
	3.Education	0.231**	0.664**	1											
	4.Monthly family income	0.111**	0.027	0.104**	1										
	5.Exercise frequency	-0.221**	-0.127**	-0.111**	-0.027	1									
	6.Duration of each exercise	-0.118**	0.158**	0.179**	0.093**	0.208**	1								
	7.Total weekly exercise hours	-0.222**	0.000	0.017	0.030	0.809**	1.000	1							
	8.Whether to use the smartphone before sleep	0.012	-0.020	-0.018	0.068*	-0.088**	0.721**	-0.099**	1						
	9.Duration of smartphone use before sleep	0.072*	0.109**	0.078*	0.048	-0.166**	-0.063	-0.093**	0.190**	1					
	10.Sleep delay due to smartphone use	0.104**	0.110**	0.116**	0.013	-0.117**	0.050	-0.081*	0.109**	0.267**	1				
	11.Sleep quality score	0.078*	0.100**	0.105**	0.035	-0.068*	0.012	-0.028	0.064	0.130**	0.198**	1			
	12.Smartphone addiction	0.117**	0.020	0.042	0.016	-0.165**	0.023	-0.160**	0.055	0.229**	0.226**	0.166**	1		
	13.Anxiety	0.170**	0.198**	0.239**	0.060	-0.142**	-0.073*	-0.105**	0.036	0.152**	0.182**	0.321**	0.288**	1	
	14.Depressive	0.113**	0.156**	0.152**	0.012	-0.074*	-0.005	-0.055	0.064	0.205**	0.183**	0.435**	0.329**	0.690^**^	1

**. Significant correlation at 0.01 level (bilateral).

*. Significant correlation at 0.05 level (bilateral).

### Association between nighttime exercise indices and variables of interest

Table 4 demonstrates the associations of nighttime exercise indices and smartphone-related health outcomes among individuals who claimed to primarily exercised at night. In the adjusted model, the frequency of nighttime exercise was negatively associated with the likelihoods of using a smartphone before sleep and sleep delay due to smartphone use, as well as the duration of smartphone use before sleep smartphone and the levels of addiction and anxiety. The duration of nighttime exercise (per time) was only negatively associated with the likelihood of using a smartphone before sleep. The total duration of nighttime exercise was negatively associated with the likelihood of using smartphone before sleep, the duration of smartphone use before sleep, and the levels of smartphone addiction and anxiety.

**Table 4:** Association between nighttime exercise programs and core variables

Outcome	VGW items	Crude model		Adjusted model	
		B/OR (95%CI)	*p*	B/OR (95%CI)	*p*
Use smartphone before sleep	Nighttime exercise frequency	**0.987(0.979, 0.995)**	**0.002**	**0.987(0.979, 0.995)**	**0.001**
	Nighttime exercise duration (per time)	**0.983(0.971, 0.996)**	**0.013**	**0.983(0.970, 0.996)**	**0.013**
	Nighttime exercise total duration	**0.994(0.992, 0.997)**	**0.000**	**0.994(0.991, 0.997)**	**0.000**
Duration of smartphone use before sleep	Nighttime exercise frequency	**-0.253(-0.364, -0.142)**	**0.000**	**-0.231(-0.344, -0.117)**	**0.000**
	Nighttime exercise duration (per time)	0.110(-0.074, 0.294)	0.242	0.112(-0.076, 0.301)	0.243
	Nighttime exercise total duration	**-0.041(-0.077, -0.005)**	**0.025**	**-0.037(-0.074, -0.001)**	**0.045**
Sleep delay due to smartphone use	Nighttime exercise frequency	**0.968(0.948, 0.988)**	**0.002**	**0.975(0.955, 0.996)**	**0.021**
	Nighttime exercise duration (per time)	1.015(0.981, 1.051)	0.388	1.014(0.979, 1.051)	0.431
	Nighttime exercise total duration	0.995(0.989, 1.002)	0.182	0.997(0.990,1.004)	0.361
Poor sleep quality	Nighttime exercise frequency	**-0.099(-0.195, -0.002)**	**0.044**	-0.069(-0.168, 0.029)	0.166
	Nighttime exercise duration (per time)	0.034(-0.125, 0.192)	0.677	0.020(-0.143, 0.182)	0.813
	Nighttime exercise total duration	-0.023(-0.053, 0.008)	0.150	-0.019(-0.051, 0.012)	0.235
Smartphone addiction	Nighttime exercise frequency	**-0.499(-0.715, -0.283)**	**0.000**	**-0.433(-0.654, -0.212)**	**0.000**
	Nighttime exercise duration (per time)	**-0.374(-0.732, -0.015)**	**0.041**	-0.326(-0.693, 0.041)	0.081
	Nighttime exercise total duration	**-0.141(-0.210, -0.072)**	**0.000**	**-0.123(-0.194, -0.052)**	**0.001**
Anxiety	Nighttime exercise frequency	**-0.335(-0.522, -0.148)**	**0.000**	**-0.223(-0.408, -0.037)**	**0.019**
	Nighttime exercise duration (per time)	-0.060(-0.370, 0.250)	0.704	-0.198(-0.505, 0.109)	0.205
	Nighttime exercise total duration	**-0.076(-0.136, -0.016)**	**0.014**	**-0.070(-0.129, -0.010)**	**0.021**
Depression	Nighttime exercise frequency	**-0.196(-0.382, -0.010)**	**0.039**	-0.131(-0.319, 0.058)	0.173
	Nighttime exercise duration (per time)	-0.076(-0.382, 0.231)	0.629	-0.141(-0.452, 0.171)	0.376
	Nighttime exercise total duration	**-0.060(-0.119, 0.000)**	**0.049**	-0.056(-0.116, 0.004)	0.069

Note: The index parameters of whether to use smartphone before sleep and whether to Sleep Delay Due to Smartphone Use are OR value

## Discussion

### General discussion

The present study aimed to investigate the proportion of Chinese university students who engage in nighttime exercise, and to evaluate the associations of nighttime exercise and smartphone use before sleep, sleep delay, sleep quality, smartphone addiction, and mental health issues.

We found that the frequency of nighttime exercise was negatively associated with the probability and duration of smartphone use before sleep and its related sleep delay. Meanwhile, it was also associated for smartphone addiction and anxiety. Similar findings were obtained for the total duration of nighttime exercise, except for sleep delay (which was non-significant).

The findings on smartphone addiction, smartphone use before sleep, and related sleep delay broadly support our first, second, and fourth hypotheses, and they are generally in line with findings of previous studies [24, 25, 71, 72]. These phenomena may be explained by the following reasons. Unhealthy smartphone use before sleep has been regarded as an addictive behavior [26], a subtype of impulse control disorders [73]. Physical exercise is known to enhance neural plasticity related to reward in key brain structures such as the dorsolateral striatum, the nucleus accumbens, and the ventromedial prefrontal cortex [74], thus mitigating the influence of impulse control. A study in 2020 confirmed that engaging in physical exercise stimulates the pituitary gland to release endorphins, enhancing pleasure and reducing discomfort when away from smartphones [75].

In concordance with earlier research, we observed that college students frequently postpone exercise to nighttime hours to accommodate their academic schedules and leisure time [76]. Contrary to our third hypothesis, however, our findings did not reveal a significant association between nighttime exercise behaviors and sleep quality. So far, some scholars believe that engaging in physical activity at night improves sleep, but there are also those who hold completely opposite opinions. For example, a study showed that university students who exercised at night experienced lower sleep quality and sleep efficiency compared to their morning-exercising counterparts [77]. Sleep hygiene guidelines also caution against engaging in high-intensity physical activities in the evening [53], because it is believed that physical exercise before sleep can cause nerve stimulation and reduce sleep quality. In contrast, a study on young males following nighttime exercise observed improved subjective feelings upon waking the next morning [78], and further evidence also suggests that engaging in moderate-intensity exercise 2–4 h before sleep may positively change sleep-related variables such as sleep delay and slow-wave sleep [79–81]. Therefore, the relationship between nighttime exercise and sleep quality remains complex and inconclusive. It is necessary to explore which side, benefits or drawbacks, is more predominant in specific scenarios.

Ultimately, we found that engagement in nighttime exercise was associated with reduced levels of anxiety, while the association with depression was not statistically significant. This result partially supports our fifth hypothesis. Previous literature has widely recognized the health benefits of physical exercise, encompassing both increased life expectancy and enhanced mental and physical well-being [82]. One possible reason is that physical exercise can stimulate cerebral microcirculation, elevate levels of monoamine neurotransmitters in the brain, accelerate blood circulation, and enhance metabolic rates, thereby attenuating sedentary behavior and improving mood [83, 84]. Additionally, social withdrawal theory can also be employed for explanation. Lack of physical activity can lead to social isolation and withdrawal, both of which are positively correlated with increased social anxiety [85].

In comparison with this study, many previous studies have provided evidence that physical exercise can mitigate symptoms of depression in both clinical and non-clinical populations [86, 87]. To explain this discrepancy, exercise intensity may offer a clue. For example, exercise can alleviate symptoms of depression by reducing inflammation, with moderate-intensity continuous exercise identified as optimal [88]. Therefore, the absence of an examination of exercise intensity may be a reason for our null results concerning depression.

In summary, our findings indicate that nighttime exercise may have numerous potential benefits related to smartphone usage issues. Considering insufficient physical activity is one of the major crises in China currently [89–91], we advocate for the reasonable utilization of leisure time at night for exercise. It is important to note that we do not endorse the irrational extension of nighttime exercise to compromise sleep duration. Instead, we recommend viewing nighttime exercise as a leisure activity within a framework that promotes health.

### Limitations

Firstly, our data was purely self-reported, introducing the possibility of reporting biases. Respondents might be reluctant to disclose unhealthy behavioral patterns or mental conditions, which could result in underestimations of smartphone addiction, depression, and anxiety levels.

Secondly, our investigation of nighttime exercise is confined to qualitative questions and does not address the specific timing of exercise, nor the time elapsed between the end of exercise and sleep delay. Moreover, the intensity of nighttime exercise is a significant uncertainty factor, and different intensities of nighttime exercise can affect sleep quality, and even have the opposite effect. Future research exploring the relationship between nighttime exercise and sleep quality should consider the time interval and intensity between the exercise and sleep behaviors.

Thirdly, ross-sectional surveys are inevitably subject to confounding factors, making it necessary to comprehensively control external variables. Currently, many social, environmental, and cultural factors are known to potentially influence physical activity and problematic smartphone use [92–94]. However, due to limitations in survey methods, we couldn’t thoroughly investigate these factors. Therefore, future research should consider more diverse survey methods to reduce the risk of bias.

Lastly, our sample consists of university students only in Chongqing City. The comparatively homogeneous environment and academic-focused stressors of this population could limit the generalizability of our findings. Subsequent research should target diverse populations to explore the relationships between exercise habits and health behaviors, so as to enrich the theoretical framework concerning nighttime exercise.

## Conclusion

The aim of this study was to investigate the impact of nighttime exercise on smartphone usage, sleep quality, and health-related behaviors. We found that nearly 70% of the participants chose to engage in physical exercise during nighttime, a choice closely related to academic pressures during daytime. Moreover, the frequency and overall duration of nighttime exercise were significantly correlated with smartphone use before sleep, the length of smartphone use before sleep, sleep latency, smartphone addiction, and anxiety. However, weaker correlations were observed between sleep quality and depression. These findings could serve as preliminary evidence supporting the notion that nighttime exercise might reduce smartphone addiction and alleviate anxiety.

## Data Availability

The data collected in this study will not be publicly available. However, the corresponding author can be contacted for de-identified data on reasonable request.
